# Supplementary material for: Causal interplay between lactose intolerance and gut microbiota: a combined bidirectional Mendelian randomization and in vivo validation study
Source: Front Nutr. 2026 Jun 1;13:1803337. doi: 10.3389/fnut.2026.1803337 (PMC13265576; doi:10.3389/fnut.2026.1803337)
Supplement: Supplementary file 1 [file Data_Sheet_1.zip › supplementary materials/Forward/leave one out cross validation/ebi-a-GCST90027593.finngen_R12_E4_LACTONAS.pdf]

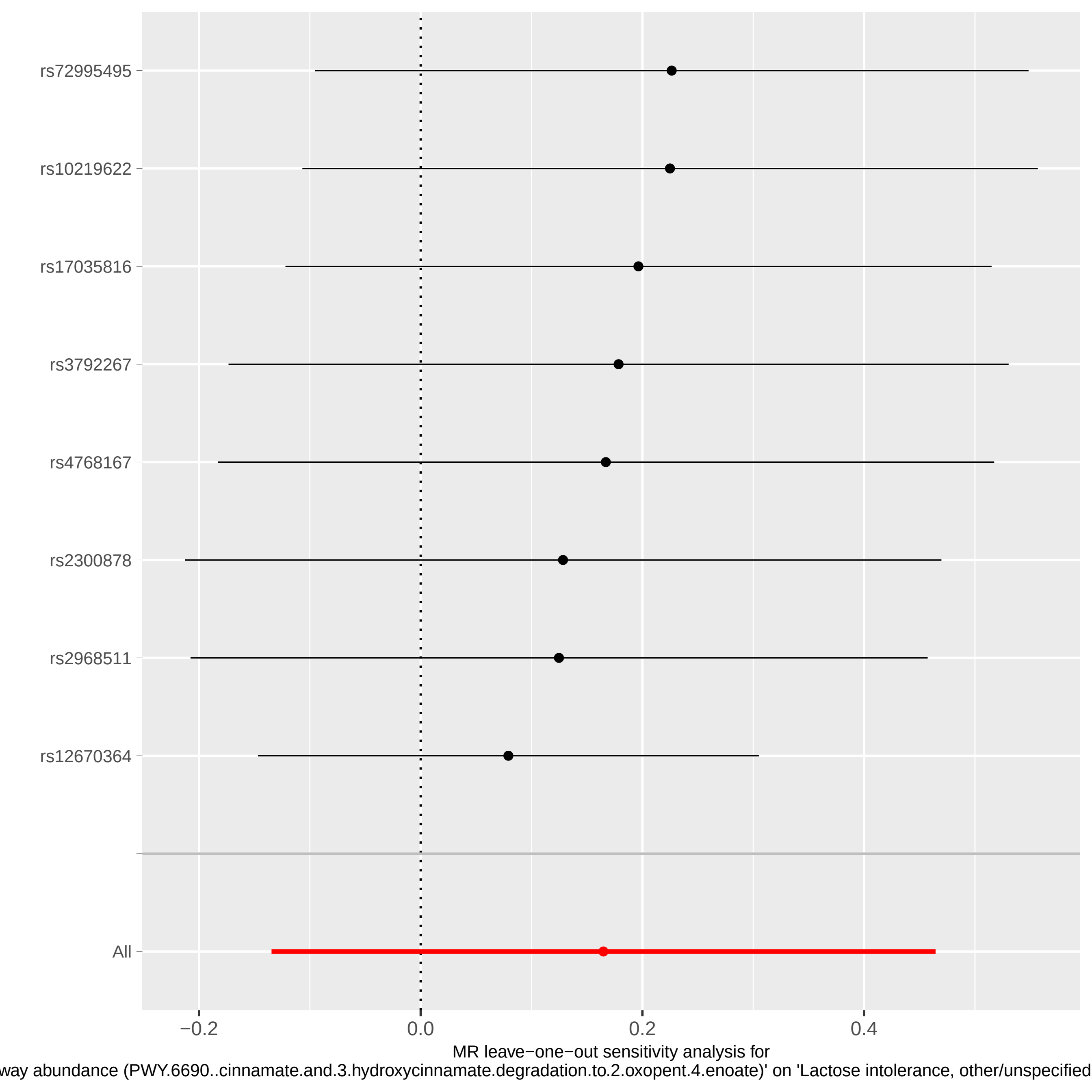
rs72995495
rs10219622
rs17035816
rs3792267
rs4768167
rs2300878
rs2968511
rs12670364
All
−0.2
0.0
0.2
0.4
MR leave−one−out sensitivity analysis for
way abundance (PWY.6690..cinnamate.and.3.hydroxycinnamate.degradation.to.2.oxopent.4.enoate)' on 'Lactose intolerance, other/unspecified
